# How do small groups make decisions? A theoretical framework to inform the implementation and study of clinical competency committees

**DOI:** 10.1007/s40037-017-0357-x

**Published:** 2017-05-22

**Authors:** Saad Chahine, Sayra Cristancho, Jessica Padgett, Lorelei Lingard

**Affiliations:** Centre for Education Research & Innovation, Schulich School of Medicine and Dentistry, Western University, London, Ontario Canada

**Keywords:** Competency-based medical education (CBME), Clinical competency committees, Group decision-making

## Abstract

In the competency-based medical education (CBME) approach, clinical competency committees are responsible for making decisions about trainees’ competence. However, we currently lack a theoretical model for group decision-making to inform this emerging assessment phenomenon. This paper proposes an organizing framework to study and guide the decision-making processes of clinical competency committees.

This is an explanatory, non-exhaustive review, tailored to identify relevant theoretical and evidence-based papers related to small group decision-making. The search was conducted using Google Scholar, Web of Science, MEDLINE, ERIC, and PsycINFO for relevant literature. Using a thematic analysis, two researchers (SC & JP) met four times between April–June 2016 to consolidate the literature included in this review.

Three theoretical orientations towards group decision-making emerged from the review: schema, constructivist, and social influence. Schema orientations focus on how groups use algorithms for decision-making. Constructivist orientations focus on how groups construct their shared understanding. Social influence orientations focus on how individual members influence the group’s perspective on a decision. Moderators of decision-making relevant to all orientations include: guidelines, stressors, authority, and leadership.

Clinical competency committees are the mechanisms by which groups of clinicians will be in charge of interpreting multiple assessment data points and coming to a shared decision about trainee competence. The way in which these committees make decisions can have huge implications for trainee progression and, ultimately, patient care. Therefore, there is a pressing need to build the science of how such group decision-making works in practice. This synthesis suggests a preliminary organizing framework that can be used in the implementation and study of clinical competency committees.

## Introduction

Competency-based medical education (CBME) emphasizes the systematic collation and analysis of multiple assessment data points to support judgments about learner progress [1–4]. Clinical competency committees are small groups entrusted with these analytic and decision-making tasks [5, 6]. These committees play a dual role, as both gatekeepers and learning supports, ensuring patient safety and guiding trainees [7–9]. Currently, however, medical education offers no theoretical model for group decision-making to inform this emerging assessment phenomenon. The implementation, monitoring, and scientific study of decision-making in clinical competency committees would be strengthened by such a shared theoretical model. This eye-opener describes one possible model, drawn from a synthesis of group decision-making literature in other domains.

A body of literature is emerging on clinical competency committees: papers that describe the purposes and rationale of clinical competency committees [7]; formal and informal guidelines that support the development of these committees [5, 10, 11]; and studies that document the processes involved [12–15]. A 2016 narrative review has highlighted the initial literature relevant to clinical competency committees [7]. Much of this review was devoted to important elements of clinical competency committees such as group membership and optimal number of participants. And while group processing was one of many themes identified in this review, it was not explored in depth. In this eye-opener, we specifically focus on understanding group processing by conducting a more in-depth review across multiple fields. Furthermore, we synthesized the literature in order to offer an initial theoretical framing of decision-making in clinical competency committees. Given the potential consequences clinical competency committees have for both learner development and patient care, we anticipate a groundswell of research on small group decision-making in the coming years as CBME is actualized [16]. A shared theoretical framework and common language will aid in understanding variations in implementation across different specialties and programs [17–20].

## Methods

This paper presents an initial theoretical framing to understand how clinical competency committees make decisions. Our purpose was to summarize and synthesize major ideas and concepts across several fields that study how small groups make decisions. Our review is non-exhaustive and is intended to be explanatory, drawing on concepts from other fields to inform a theory of how clinical competency committees might make decisions. Through a three-step process, we drew on narrative review methodology and qualitative thematic analysis to develop and refine major groupings of papers and concepts [21]. In the first step, authors (SC and JP) met and identified research fields to focus the search: organizational psychology, business, Mmedicine, and education were selected as starting points. Recognizing that there is a breadth of content and that some concepts may not be published in academic journals, we expanded the search to include conference materials and books. We also included the grey literature, which are publications that can be produced by government and organizations that may not be indexed by academic journals [22]. After the initial search, SC & JP met to discuss the recurring concepts identified in the literature. In step 2, SC & JP conducted a more in-depth search and developed a list of sources (books and papers) that elaborated these concepts. In step 3, SC & JP reviewed the sources and, by comparing the content of the papers, developed thematic groupings to represent the findings of the current paper.

## Results: a theoretical framework for group decision-making

**Fig. 1:**
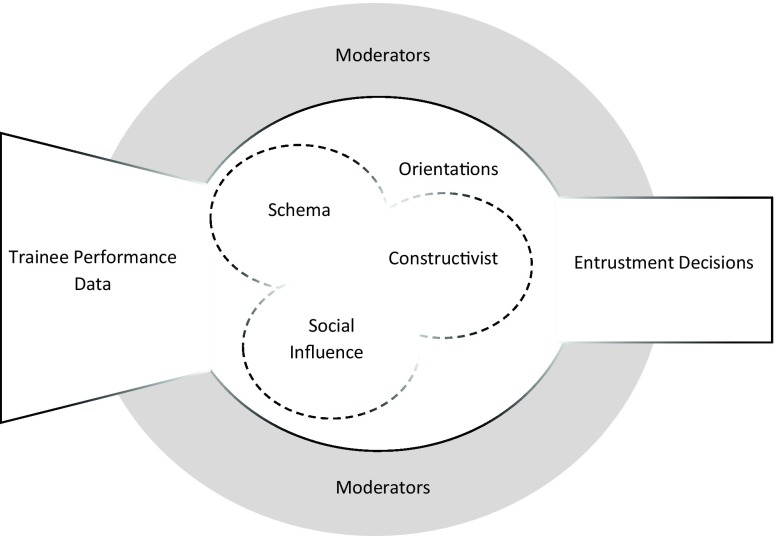
Theoretical framework for clinical competency committee decision-making

We synthesized existing group decision-making concepts into three *orientations* towards group decision-making: schema, constructivist, and social influence. As a theoretical framework, these three orientations reflect potential models of decision-making that clinical competency committees could use when they review trainee performance data. While they are presented independently, in practice these orientations are dynamic and can overlap with one another. Additionally, *moderators*, such as guidelines, time pressures and leadership, can influence the orientation of each clinical competency committee and the subsequent decisions that are made. This initial theoretical framework of *orientations* and *moderators* is presented in the context of how clinical competency committees may make decisions in Fig. 1.

### Schema orientations

Schemas are organized, algorithmic processes which provide a structure for integrating new information and evaluating it based on specific behavioural tasks [23]. In terms of decision-making, a schema orientation frames decision-making in groups as a well-structured, task-focused process. It typically views groups and the decisions they have to make in formulaic terms [24]. Some of these models are general, describing the processes that any group would go through to make a decision, while some are more specifically tailored to certain environments [25]. A few even provide algorithms to predict the best or most likely choice a group will make [26–28].

One of the earliest and most prominent approaches within the schema orientation is social decision scheme theory, which is essentially a rule or procedure (complete with a formula) that determines how groups combine their own preferences to achieve a collective group choice [24, 29, 30]. According to Strasser, there are four basic elements to social decision scheme theory [29]:

1. Individual preferences: where individual group members’ preferences are identified
2. Group preference compositions: where the group preference is calculated using a formula or algorithm
3. Patterns of group influence: where a ‘decision scheme’ matrix is developed that supports the decision-making
4. Collective responses: where the group determines a response based on the decision.

Alternative models to social decision scheme theory, which also focus on aggregating information, include social combination schemes, social judgment schemes, and median-based models [31]. These models use various methods to aggregate the preferences of individual group members to form a single group-level preference.

Within the schema orientation, there are also non-aggregation approaches which describe the general structure of the decision-making process. The functional model is an approach to group decision-making that focuses on the systematic processing of group decisions. It claims that in order to make the best possible decision, there are a set of requirements (problem analysis, establishment of evaluation criteria, generation of alternative solutions, evaluation of positive consequences of solution, and evaluation of negative consequences of solutions) that the group must participate in over the course of their discussion [32, 33]. Similarly, the groups as information processors approach argues that groups process information much like individuals do; they focus their attention on specific information and then process, encode, store and retrieve it to make a decision [34]. While the previous two models provide general frameworks for how groups make decisions, there are also examples of very specific models, such as the analytic hierarchy process, which was developed for use in healthcare settings. It is designed for groups to follow an eight-step decision-making process which includes defining the problem, evaluating the problem and the group’s understanding of it, and the final choice made by the group [35].

Overall, schema orientations offer a well-defined, and easily measured approach to decision-making by breaking the process into quantifiable parts and sometimes providing highly specific steps to be followed. However, a drawback of this orientation is its assumption that people and groups take a mechanistic approach to decision-making.

### Constructivist orientations

When group decision-making is viewed from a constructivist paradigm, ‘processing involves an active search for understanding in which incoming experience is reorganized and integrated with existing knowledge’ [36, p. 156] Constructivist orientations differ from schema orientations as they focus on the group’s construction of their shared understanding, instead of focusing on the specific steps that groups follow during the decision-making process.

Central to constructivist orientations is the idea of a shared mental model. A shared mental model is the organized knowledge shared by a group of team members [37]. This knowledge can represent their shared understanding of the group’s task and/or their shared understanding about the group itself and its members [30, 31]. Similarly, the group’s knowledge about the distribution of information among the group members, i. e., knowing which group members know what, reflects their collective understanding and awareness of the group itself [38]. The state of a group’s shared mental model and self-knowledge can influence how well groups make decisions by influencing how well they are able to share and discuss information.

The capability of a group to construct a decision together, which would be better than any of its individual member’s decisions, is referred to as synergy [39]. Theoretically, the higher the group’s synergy, the better their decision outcome. A high degree of synergy is important in situations where there is a *hidden profile,* which is when individuals in the group have information that other group members do not have [39, 40]. In a highly synergistic group, this hidden information is shared appropriately which supports the final collective decision [41]. Time is spent on deliberating and processing information as it is exchanged across group members and there is a shared understanding of the implications of each decision. Group members also discuss and deliberate alternatives that the group may not have initially conceived [41].

Across the varied models and approaches in the constructivist orientation, there is a focus on how and how well groups share information and work together during the decision-making process. These orientations generally focus on a holistic, socially-orientated approach to decision-making, paying close attention to the group’s collection, and hence construction, of knowledge. They typically argue that there is a shared mental model within the group whereby group members collectively understand the purpose, implications, and potential alternatives of their group decision. According to the constructivist approach, a strong group is one that shares their information efficiently and effectively in order to reach an ideal decision outcome.

### Social influence orientations

Social influence is based on the idea that decisions in groups may not be as neatly organized as they are conceptualized in schema orientations, nor may they have a shared and coordinated effort to produce the best decisions as conceptualized in the constructivist orientations. Rather, social influence orientations focus on how individuals within a group can be influenced and how perspectives may change based on social pressures [42]. Researchers have been attempting to formulate how communication and social judgments interact when people make decisions [43]. Researchers have also been perplexed by how individuals conform within group contexts [44, 45]. Groupthink theory suggests that very smart individuals working together often concede to a collective decision just for the sake of consensus rather than carefully working out the best choice [46, 47].

Social influence orientations attend closely to the processes used in the group to deliberate members’ varied perspectives. Three processes have received particular attention [48]:

1. Argumentation processes: where group members discuss and justify their pre-conceived decision, yet are open to being influenced by the knowledge voiced by other members
2. Comparison processes: where group members voice and share their decision preferences in comparison to other member’s preference, yet are open to change
3. Compliance processes: where group members voice the decision preferences they believe will gain them social approval in their group.

Engagement in any of these processes is activated by the context of a given situation and influences the members’ subsequent message to the group. This in turn affects subsequent discussion and communication. Through these processes, multiple communication feedback loops amongst members eventually result in a decision. Social hierarchy and issues of power are important considerations in social influence orientations. For instance, if an individual is in a group with several of their supervisors, they might be more inclined to engage in compliance processes which will influence how they communicate with the group. As a result, the decision the group makes may not be the most adequate but rather the result of strong social influences. Being aware of how the social environment influences decisions can allow groups to put strategies in place that are aimed at reducing such negative consequences.

Social influence orientations frame decision-making as an active process where individuals are expected to argue, compare or comply with majority choice. Models such as structuration and bona fide groups focus on the societal context that the group exists within, suggesting the rules a group develops for its decision-making procedures and the way its members interact with one another are dependent on both immediate and broad societal influences [49]. This could apply to the micro social environment, such as the professional and personal relationships of the members of the group as well as to the macro environment of the culture of the company, city, or society at large. Other models, such as social comparison theory [24] and social identity theory [49], focus on how ‘choice shift’ occurs – i. e., which social interactions within the group persuade or influence members to change their decision choice. Methods have also been developed which attempt to account for socially relevant communication influences on decision outcomes [48, 50].

Social influence orientations are more complex than the schema or constructivist orientations. They focus on implicit power dynamics and the rules of engagement in process, where the lines of influence may not be explicit and choice shift is expected and ongoing. In contrast to schema and constructivist orientations, social influence orientations assume that groups will not necessarily come to the correct or best decision outcome. Rather, they are likely to be influenced by internal social and external societal pressures which might lead them to make consensual but potentially inadequate choices.

### Decision-making moderators

Regardless of orientation, the literature acknowledges that all small group decision-making processes are subject to a set of moderators that can influence the decision outcome [51]. Moderators that can influence decision outcomes are structural elements such as guidelines, meeting times, and scheduling. For example, while basic guidelines can be useful in organizing a group’s task, if a guideline is overly prescriptive or too complicated, it may cause additional confusion or it may be completely ignored by the group as it is viewed as more of a hindrance than a help [52]. Additionally, stressors such as having a strict time constraint can result in a greater desire for uniformity and groups may choose an option that presents itself quickly or which is proposed by the pushiest group members [31]. Individuals who are more open and talkative may have a greater influence on the decision-making process than those who are shy or reserved, especially when there is a lack of information [53]. Among these moderators, potentially the most influential is the group leader, whether selected or emergent. Leaders are enormously influential in terms of how the group understands and undertakes its tasks [38]. And when a leader is responsible for the decisions, they may give their own position more weight than other group members [54].

In the theoretical model presented in Fig. 1, we have positioned moderators as part of the context to reflect their substantial role regardless of the orientation used to understand a group’s decision-making process. We speculate that the degree of each moderator’s impact can differ from one decision to the next and from group to group as a result of orientation and group composition.

## Discussion

In medical training, clinical competency committees are a relatively new dimension of assessment and therefore the nature of their decision-making practices is largely unknown. While the 2016 narrative review by Hauer et al. [7] provided an overview of the major elements of clinical competency committees such as group composition, ideal group size, processing and time pressure, in this eye-opener, we have focused in depth on group processing as we attempt to synthesize and translate for medical education the breadth of knowledge on small group decision-making. The schema, constructivist and social influence orientations we have described are a synthesis of models, approaches and frameworks of decision-making from multiple fields, including business and management, organizational psychology, healthcare and education.

The orientations can be understood as offering progressively more complex views of the group decision-making process, and each offers different affordances for implementation and scholarship regarding clinical competency committees. The schema orientation offers algorithmic processes and defined steps, the constructivist orientation foregrounds shared goals and mental models, while the social influence orientation attends to the often tacit and unpredictable power relations complicating the interactions among individuals in decision-making groups. Importantly, these orientations are not mutually exclusive. Group processes may well include clear steps (*schema*), shared goals (*constructivist*) and tacit influences due to hierarchy (*social influence*); accordingly, a framework for understanding these processes should be alert to dimensions from each of these orientations and how they may be interacting in clinical competency committee behaviours. Thus, our synthesis does not privilege one orientation over the other, or recommend one as more relevant for clinical competency committee implementation or scholarship efforts. Rather, we would contend that clinical competency committees are likely to be best understood through a rich set of theoretical lenses, as they will undoubtedly involve some simple algorithms (e. g., a set of steps for reviewing and weighting available trainee assessment data to come to a decision), some shared mental models (e. g., a collective understanding of the nature and implications of the particular professional activity they are deciding whether to entrust a trainee for), and some social influence processes for negotiating meaning (e. g., compliance processes in clinical competency committees which combine senior and more junior faculty members). Finally, our inclusion of moderators in the framework reflects the need to better understand how these influence not only the decision but also the dynamic processes by which a clinical competency committee will come to its decision.

## Conclusion

Much attention is being paid to milestone and entrustable professional activity (EPA) articulation in CBME, but the decision-making processes surrounding trainees’ progress has received very little focus. There is a pressing need to build the science of how CBME decision-making works in practice, when a group of clinicians must come to a shared decision about trainee progression. How clinical competency committees make decisions, and the potential consequences of these, are currently unknown. As medical education moves increasingly towards this new model of small group decision-making for critical decisions about entrustment in training, we require a shared language and framework for coherently organizing a range of activities, from clinical competency committees creation and training, to evaluation and empirical study. The framework of orientations and moderators described in this eye-opener exposes the medical education community to some of the key knowledge about group decision-making from other fields. We offer this framework as a basis for advancing the work of implementation and scholarship around clinical competency committees.
